# Therapeutic Hypothermia Reduces Peritoneal Dialysis Induced Myocardial Blood Flow Heterogeneity and Arrhythmia

**DOI:** 10.3389/fmed.2021.700824

**Published:** 2021-07-30

**Authors:** Sanjay R. Kharche, Sandrine Lemoine, Tanya Tamasi, Lisa Hur, Aaron So, Christopher W. McIntyre

**Affiliations:** ^1^Kidney Clinical Research Unit, Lawson's Health Research Institute, Victoria Hospital, London, ON, Canada; ^2^Department of Medical Biophysics, Schulich School of Medicine and Dentistry, University of Western Ontario, London, ON, Canada; ^3^Imaging Program, Lawson Health Research Institute, London, ON, Canada

**Keywords:** peritoneal dialysis, therapeutic hypothermia, computed tomography imaging, computational cardiology, arrhythmia

## Abstract

**Background:** Moderate therapeutic hypothermia (TH) is a well-recognized cardio-protective strategy. The instillation of fluid into the peritoneum provides an opportunity to deliver moderate hypothermia as primary prevention against cardiovascular events. We aimed to to investigate both cardiac perfusion consequences (overall blood flow and detailed assessment of perfusion heterogeneity) and subsequently simulate the associated arrhythmic risk for patients undergoing peritoneal dialysis (PD) induced TH.

**Methods:** Patients underwent high resolution myocardial perfusion scanning using high resolution 256 slice CT scanning, at rest and with adenosine stress. The first visit using the patient's usual PD regimen, on the second visit the same regime was utilized but with cooled peritoneal dialysate at 32°C. Myocardial blood flow (MBF) was quantified from generated perfusion maps, reconstructed in 3D. MBF heterogeneity was assessed by fractal dimension (FD) measurement on the 3D left ventricular reconstruction. Arrhythmogenicity was quantified from a sophisticated computational simulation using a multi-scale human 3D ventricle wedge electrophysiological computational model.

**Results:** We studied 7 PD patients, mean age of 60 ± 7 and mean vintage dialysis of 23.6 ± 17.6 months. There were no significant different in overall segmental MBF between normothermic condition (NT) and TH. MBF heterogeneity was significantly decreased (−14%, *p* = 0.03) at rest and after stress (−14%, *p* = 0.03) when cooling was applied. Computational simulation showed that TH allowed a normalization of action potential, QT duration and T wave.

**Conclusion:** TH-PD results in moderate hypothermia leading to a reduction in perfusion heterogeneity and simulated risk of non-terminating malignant ventricular arrhythmias.

## Introduction

Patients receiving peritoneal dialysis (PD) are faced with the equivalent survival challenges as patients treated with hemodialysis (HD), both in terms of the rate and dominance of cardiac sudden death as the main modality of cardiovascular mortality (1, 2). Beyond classic atherosclerotic disease (most common factor in the non-kidney disease population), very high mortality rates in PD patients are a result of the combination of additional factors such as metabolic stress, vascular calcification, myocardial fibrosis and endothelial dysfunction. At present there are no therapies identified able to provide primary prevention of sudden cardiac death events in patients receiving dialysis. Conventional therapies developed within the general population (such as statins and antiplatelet therapies) are ineffective in reducing cardiovascular mortality in either PD or HD patients. Furthermore, the risk/benefit considerations of device-based interventions are rather different in patients receiving both forms of dialysis (3–5) and currently not recommended as primary prevention in patients receiving PD (6).

Alteration of microcirculation plays a fundamental role in development of these cardiovascular outcomes. Microcirculatory function is important in determining the overall blood flow in the myocardium (especially when under demand) but it is increasingly recognized that the pattern of perfusion may be important in determining the underlying electrophysiological properties of cardiac muscle. The microcirculation of the normal myocardium is heterogeneous during rest. This heterogeneity of perfusion is an inherent and functionally significant property of microvascular network. However, when myocardial blood flow increases, oxygen extraction efficacy can be improved by homogenizing capillary flow. An increase of heterogeneity of myocardial perfusion, *per se*, is a marker of coronary endothelial dysfunction and is associated with coronary atherosclerosis, independently from traditional regional myocardial perfusion defects (7). Lu and co-workers demonstrated increased heterogeneity of stress myocardial blood flow (MBF) in hypertrophic cardiomyopathy patients is associated with an increased risk of ventricular arrhythmias (8). Cellular level experiments reveal perfusion heterogeneity disrupts the passage of myocardial depolarization fragmenting the passage of the electrical activity through the heart and resulting in ventricular re-entrant circuits (9). Increasingly heterogenous myocardial perfusion at the microvascular level has been proposed as a mechanism in arrhythmia and therefore of sudden cardiac death.

Therapeutic induction of mild hypothermia in animal studies has been shown to improve myocardial microvascular integrity under conditions of stress (10). Reduction of temperature by using cooled dialysis is a promising cardio-protective intervention in HD patients (11–13) and is currently being tested in a large-scale randomized controlled trial of 14,000 patients (MY TEMP study- NCT02628366) (14). In PD, the peritoneum provides a large surface area for thermal transfer, can be performed at night; providing potential coverage a substantial part of the 24 h period and in particular the circadian vulnerability to sudden cardiac death.

We hypothesized that moderate therapeutic hypothermia (TH) might have the potential to provide primary cardio-protection in PD patients by effecting overall myocardial blood flow, ischemic tolerability and perfusion heterogeneity influencing cardiac arrhythmic potential. The aim of this proof of principal study was to investigate both cardiac perfusion consequences (overall blood flow and detailed assessment of perfusion heterogeneity) and subsequently simulate the associated arrhythmic risk for patients undergoing PD induced TH.

## Methods

### Patients

Seven participants were recruited from the Renal Program at London Health Sciences Center. Inclusion criteria were patients receiving daily PD treatment at home, older than 18 years and residual renal function ≤ 750 mL per 24-h period. Exclusion criteria were previous adverse reaction to intravenous contrast, allergy to adenosine, exposure to PD for <90 days prior to recruitment, ongoing spontaneous bacterial peritonitis (SBP), severe heart failure (New York Heart Association grade IV), cardiac transplant recipients and mental incapacity to consent. All patients gave written informed consent. This study was conducted according to the principles of the Declaration of Helsinki, with appropriate ethics committee approval (CRIC: R-16-012; REB approval number: 107280, NCT NCT04394780).

### Study Design

**Figure 1:**
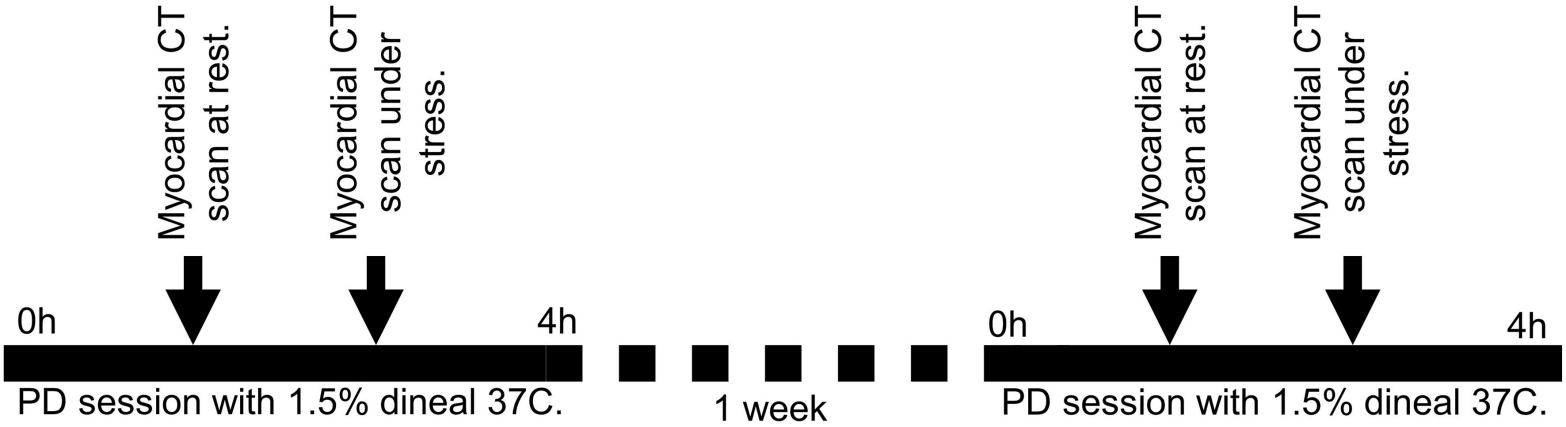
Design of the study. Patients underwent cardiac CT scans after a normothermic dialysis (NT) – 37°C (visit 1) and 1 week later, they underwent repeat CT scans after a cooled 4 h PD (TH) – 32°C (visit 2). At each visit, patient hearts were CT scanned twice for myocardial perfusion. The first scan was performed at rest; the second scan was performed 10 min after intravenous administration of adenosine to provide pharmacological stress.

We conducted a single center pilot interventional study (see Figure 1). Patients underwent cardiac CT scans after a normothermic dialysis (NT) (visit 1) and 1 week later, they underwent repeat CT scans after a cooled 4 h PD (TH) (visit 2). At each visit, patient hearts were CT scanned twice for myocardial perfusion. The first scan was performed at rest; the second scan was performed 10 min after intravenous administration of adenosine to provide pharmacological stress. Adenosine was intravenously administrated at 140 mcg/kg/min with an infusion pump, before stress myocardial perfusion acquisition was taken at 3 min into adenosine infusion.

The NT dialysate was warmed to 37°C and the cooled dialysate temperature at 32°C. Dialysate temperature was achieved using a dialysate warming cabinet (Enthermics, Wisconsin, USA). The patient's oral temperature was monitored continuously during the PD session as an indicator of core temperature.

Systemic blood pressure was recorded during each PD session (Supplementary Figure 1). Patients' characteristics, medical history and medication were recorded on visit 1. Between the two visits (no more than 2 weeks), patients continued their clinical PD prescriptions and associated medications. Patient blood samples were obtained before and after each PD session on each visit.

### Cardiac CT

#### Imaging Protocol

All studies used a 256-row GE Healthcare Revolution CT scanner with a prospective ECG gating acquisition protocol in a supine patient position: 22–25 axial scans covering 8 cm of the heart triggered at every 1–2 mid-diastole (75% R-R interval) at 100 kV tube voltage, 100 mA tube current and 280 ms gantry rotation speed with breath-hold. Dynamic contrast-enhanced (DCE) images at 5 mm slice were generated using partial-scan data collected from 180° + fan beam angle with 100% adaptive statistical iterative reconstruction (ASiR-v, GE Healthcare).

#### Myocardial Perfusion Maps

*Myocardial perfusion maps* were performed based on a model-based deconvolution approach described previously (15). Each slice of the myocardial perfusion maps was sub-divided into six standard myocardial segments in approximate horizontal long-axis according to the American Heart Association segmentation model (16) to remove non-myocardial tissue signals. The left ventricle (LV) was manually extracted (or segmented) from the myocardial BF maps to reconstruct 3D LV blood flow (BF) spatial distributions. The reconstruction was stored in a structured array of 0.5 (X) × 0.5 (Y) × 5 (Z) mm^3^ array as previously described (17) to give the appropriate high spatial resolution.

#### BF heterogeneity

*BF heterogeneity* was assessed by fractal dimension (FD) measurement on a 3D LV reconstruction as previously described (17, 18). Fractal dimensions (FD) is a strong measure of BF heterogeneity, where BF = 1.5 means a complete heterogeneity and BF = 1 a homogeneity in perfusion.

#### Myocardial Perfusion Reserve

*Myocardial perfusion reserve (MPR)* was calculated as the ratio of the mean stress to rest myocardial blood flow value.

### *In silico* Electrophysiological Computational Model

#### Cell Model

We adapted for this study the cardiomyocyte cell model developed by Ten Tusscher et al. (19). It allows simulation of electrical excitations in the human ventricle's epicardial, mid-myocardial, and endocardial regions based on key ion current parameter fine tuning (13). The cell level effects of TH were implemented based on experimental data and are fully described as Supplementary Material. This robust human ventricle cardiomyocyte cell was incorporated into novel a 3D transmural human ventricle wedge as described below.

#### 3D Transmural Human Ventricle Wedge

We developed a multi-scale *in silico* human ventricle electrophysiological model to predict electrical modification of heart through a simulated ECG. Based on our experimental CT data, we developed a 3D slab representing a large part of the left human ventricle where A virtual ECG electrode was placed 3 cm outside of the wedge as shown in Supplementary Figure 1. The electrode permitted calculation of the pseudo-ECG based on electrical activity in the wedge. The wedge was used to simulate effects of PD on ventricular ECG and activation patterns and to emphasize modifications of ECG provided by hypothermia. The detailed method is provided in Supplementary Material.

### Statistical Analysis and Sample Size Considerations

Findings from this study were expected to be essentially descriptive and provide essential preliminary data of the existence of biological effect to justify further appropriately powered studies. Given the enormous granularity of data provided by the multiple scans of each patient (with perfusion data available down to a resolution of only a few millimeters), intense complexity of performing these studies and limitations on recruitment brought abought by the challenges inherent we selected a convenience sample approach of seven subjects. All descriptive and basic statistical analysis was performed using Prism. Descriptive statistics for continuous variables were tested for normality and summarized using mean ± SD. Discrete variables were summarized as proportions. Median (min-max) BF and heterogeneity were compared between NT and TH using a non-parametric paired test (Wilcoxon test). Statistical significance was defined as *p* < 0.05. Computational methods are described above or in Supplementary Material. No data existed to provide any guidance on power calculation.

## Results

### Patient Characteristics

Mean age was 60 ± 7 years, 57% female. Mean PD vintage was 23.6 ± 17.6 months. BMI was 29.2 ± 6.4 kg/m^2^, urinary residual volume was 400 ± 300 mL. Only 1 patient had known ischemic heart disease. Documented causes of kidney disease included diabetes and/or hypertension for 2 patients, one for glomerulonephritis, one for interstitial nephritis and 3 patients had hereditary nephropathy. All characteristics are summarized in Table 1. We found no difference between bloodwork results in the patients between their 2 visits. Six patients were in automated peritoneal dialysis and 1 in continuous ambulatory peritoneal dialysis. Blood test results in Table 2. Peritoneal dialysis characteristics are described in Table 3.

**Table 1:** Patients' characteristics: anthropometrics values, medical history and medications.

	**Before NT PD**	**After NT PD**	**Before TH PD**	**After TH PD**
Sodium (mmol/L)	137 ± 3.9	134 ± 3.4	138 ± 3.5	134 ± 3.6
Potassium (mmol/L)	4.2 ± 0.8	4.3 ± 0.9	4.1 ± 0.7	4.1 ± 0.6
Chloride (mmol/L)	95 ± 4	94 ± 4	95 ± 3	94 ± 3
Bicarbonates (mmol/L)	24.1 ± 2.8	23.7 ± 2.5	25.3 ± 2.9	25.6 ± 2.1
Creatinine (umol/L)	872 ± 268	871 ± 279	821 ± 323	801 ± 315
Albumin (g/L)	34.9 ± 4.2	30.9 ± 3.8	35 ± 3.5	31 ± 12.2
Calcium (mmol/L)	2.2 ± 0.9	2.1 ± 0.2	2.2 ± 0.2	2.1 ± 0.1
Phosphate (mmol/L)	2.0 ± 0.4	1.9 ± 0.3	1.9 ± 0.4	1.76 ± 0.3
Glucose (mmol/L)	5.3 ± 1.3	5.1 ± 1.3	6.4 ± 2.6	5.8 ± 1.9
Troponine-T(ng/L) (High-sensitivity)	74.6 ± 43.5	68.6 ± 38	80.5 ± 57	55 ± 35
CRP (mg/L) (High sensitivity)	4.5 ± 3.2	4.2 ± 2.9	5.4 ± 3.6	4.2 ± 2.9

*Values are expressed as mean ± Sd or number (%)*.

**Table 2:** Bloodwork collections before and after peritoneal dialysis PD for normothermic (NT) and therapeutic hypothermia (TH) conditions.

**Characteristic**
Age (years)	60 ± 7
Female (%)	4 (57)
Weight (Kg)	77 ± 23
Height (cm)	163 ± 11
BMI (kg/m^2^)	29.2 ± 6.4
Urinary residual Volume (mL)	400 ± 300
**Medical history**
PD vintage (months)	23.6 ± 17.6
Ischemic heart disease (%)	1 (14)
Current or ex-smoker (%)	2 (29)
Peripheral vascular disease (%)	1 (14)
**Medication**
Treated hypertension (%)	6 (86)
RAAS antagonist (%)	5 (71)
β-blocker (%)	4 (43)
Statin use (%)	5 (71)
Phosphate binder *Calcium containing* (%)	5 (71)
Phosphate binder *Non calcium* (%)	1 (14)
Erythropoiesis-stimulating agent (%)	6 (86)
Vitamin D analog (%)	4 (43)

*Results are expressed as mean ± standard deviations. Comparisons between pre PD with NT and HT or post PD with NT and HT were performed with t paired test. CRP (C-reactive protein)*.

**Table 3:** Peritoneal dialysis characteristics.

**Patient number**	**APD/CAPD**	**Number of exchange**	**Dialysate**
Patient 1	APD	3	Alternation of Dianeal
Patient 2	APD-NIPD	3	Dianeal 2.5%
Patient 3	CAPD	4	Dianeal 1.5% and Extraneal 7.5%
Patient 4	APD	3	Alternation of Dianeal
Patient 5	APD-IPD	3	Alternation of Dianeal and extraneal
Patient 6	APD-IPD	3	Alternation of Dianeal and extraneal
Patient 7	APD	3	Alternation of Dianeal and extraneal

*APD, automated peritoneal dialysis; CAPD, continuous ambulatory peritoneal dialysis; IPD, intermitant peritoneal dialysis; NIPD, nocturnal intermittent peritoneal dialysis*.

### Temperature and Hemodynamics Monitoring

Cooled dialysate was tolerated well by all patients. No systemic symptoms of cold or localized drain pain were reported, despite being actively interrogated for. Median core body temperature during the PD session was not different between NT [36.5 (35.7–36.6)] and TH [36.5 vs. 36.5 (36.1–36.8°C, *p* = 0.578)] (Supplementary Figure 3A).

Median MAPs were significantly higher in TH group (92 mmHg in NT vs. 110 mmHg in TH, *p* = 0.04) during PD sessions (Supplementary Figure 3B). Hemodynamics remained stable during the PD session for the NT and the TH group. Median heart rate (HR) was not different between NT and TH group [69 vs. 62, *p* = 0.84] respectively (Supplementary Figure 3C).

### Gross Myocardial Blood flow and Myocardial Reserve

**Figure 2:**
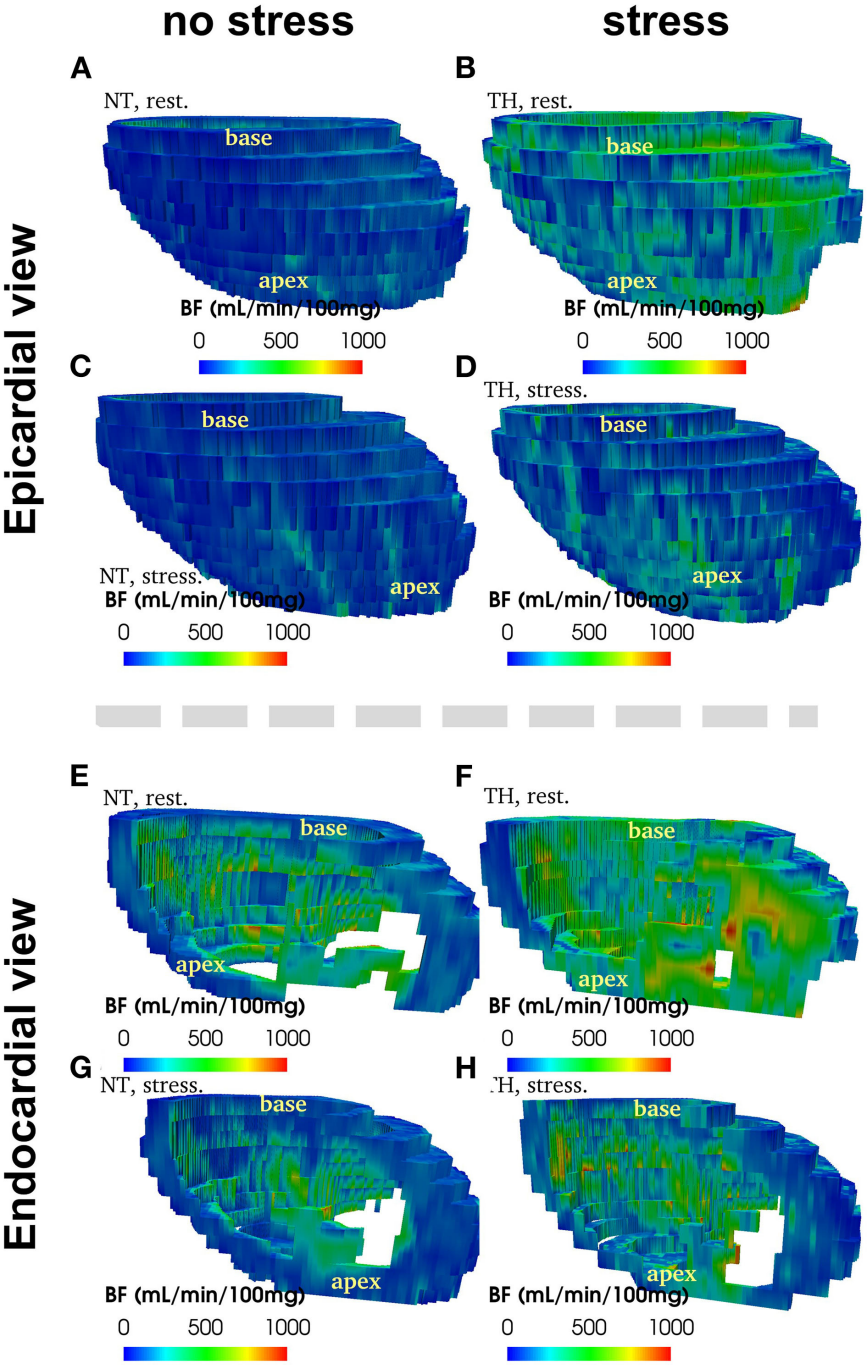
Example of 3D reconstruction of blood flow (in mL/min/100 g) segmented from CT images, measured at rest and at stress after adenosine injection, in normothermic (NT) and therapeuric hypothermia (TH) conditions for patient no. 3.

**Figure 3:**
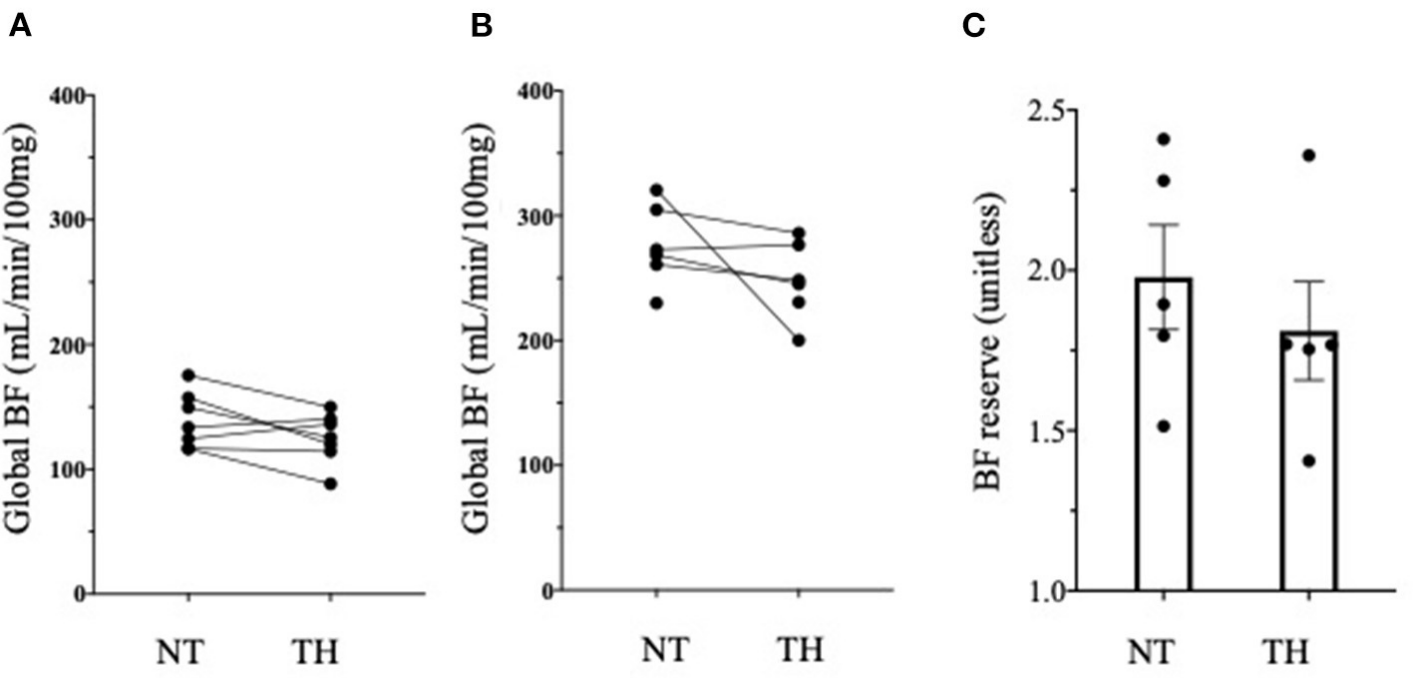
Myocardial blood flow (BF) and myocardial reserve. **(A)** We found no difference in Myocardial BF expressed in mL/min/100 g between normothermic (NT) and therapeutic hypothermia (TH) at rest using Wilcoxon test. **(B)** We found no difference in Myocardial BF expressed in mL/min/100 g between normothermic (NT) and therapeutic hypothermia (TH) at stress using Wilcoxon test. **(C)** We found no difference in myocardial reserve between NT and TH conditions.

Figure 2 provide an example of 3D reconstruction of left ventricle from where BF and fractal analysis was measured. We showed no statistical difference in overall or segmental myocardial BF between NT and TH at rest [133 (116–175) vs. 125 (88–149), *p* = 0.15, respectively] or after stress [271 (229–320) vs. 246 (200–236), *p* = 0.12, respectively] (Figures 3A,B). We found no significant difference overall or segmentally for myocardial reserve mobilization between NT and TH (189 vs. 176%, *p* = 0.06) (Figure 3C).

### Blood Flow Heterogeneity

**Figure 4:**
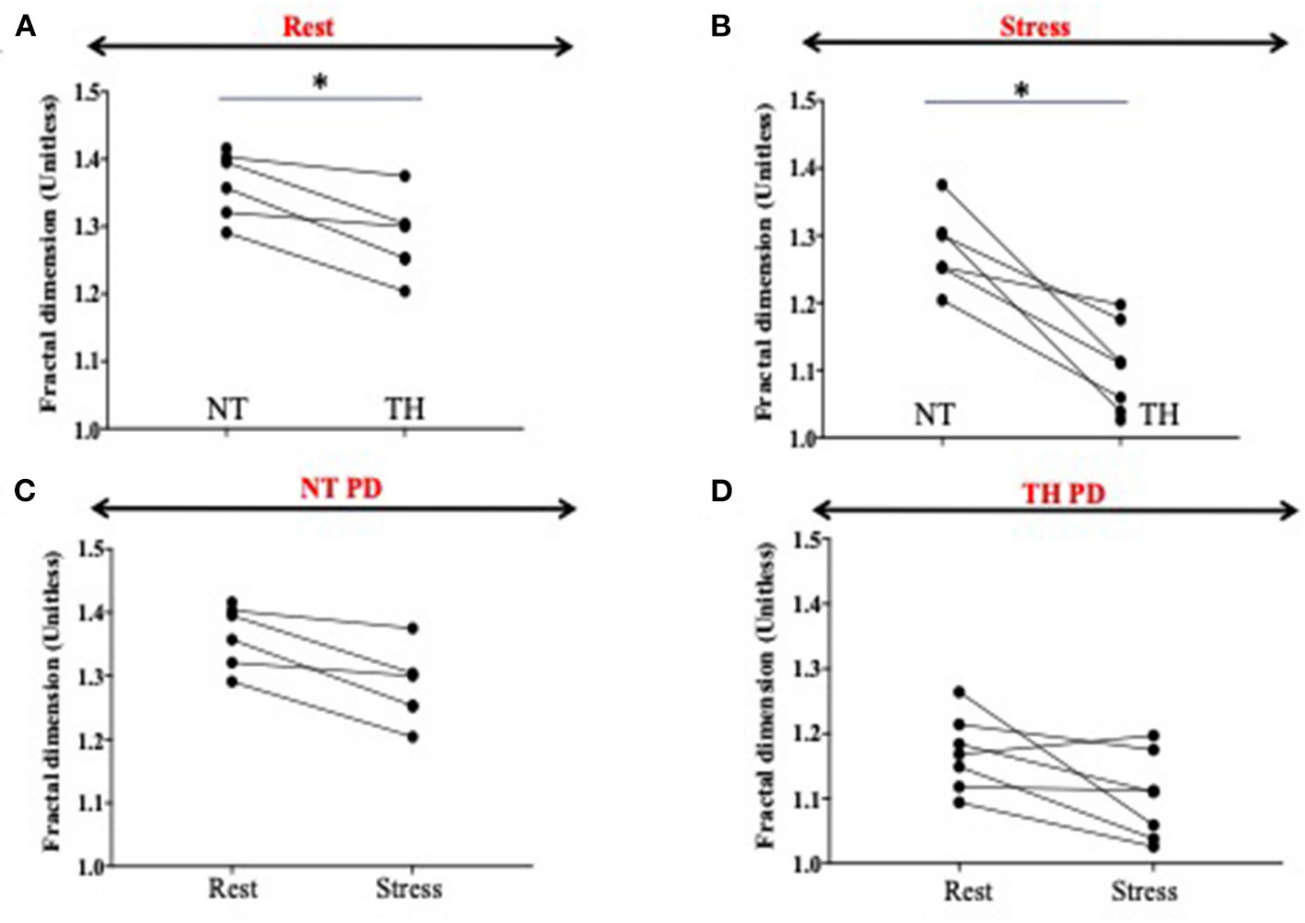
Fractal dimension as surrogate of heterogeneity. Fractal dimensions (FD) is a strong measure of blood flood (BF) heterogeneity, where BF = 1.5 means a complete heterogeneity and BF = 1 a homogeneity in perfusion. **(A,B)** Heterogeneity is significantly decreased (−14%, *p* = 0.03) at rest in the therapeutic hypothermia (HT) group compared to the normothermic (NT) group. We showed also a significant decrease of heterogeneity during the stress (−14 %, *p* = 0.03) in the HT compared to the NT group. **(C,D)** We showed a significant decrease of heterogeneity between rest and stress only in the HT group (−6%, *p* = 0.04). The * represents significance.

Heterogeneity was significantly decreased (−14%, *p* = 0.03) without pharmacological stress in the TH group compared to the NT group. We showed also a further significant decrease of heterogeneity during the stress (−14%, *p* = 0.03) in the TH compared to the NT group (Figures 4A,B). There was significant decrease of heterogeneity between rest and stress only in the TH group (−6%, *p* = 0.04) (Figures 4C,D).

### Simulated Electrophysiological Effects of Temperature on ECG and Tachycardia Persistence

#### Effects of Cooling on Cardiomyocytes

**Figure 5:**
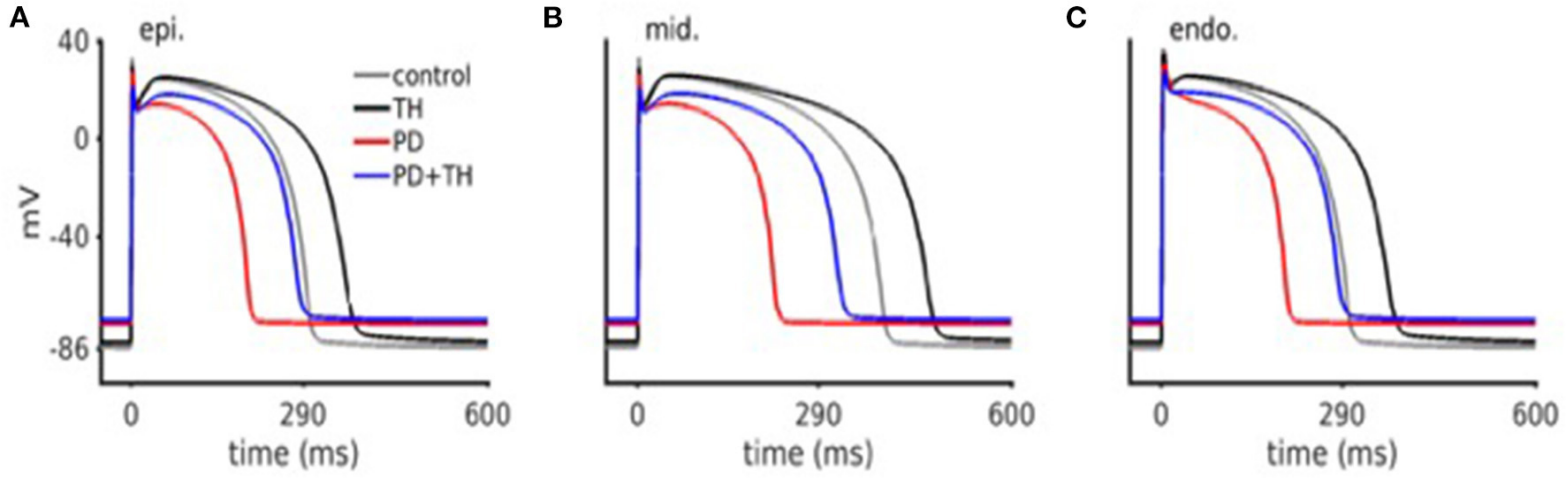
*In silico* cardiac action potential. Ischemic action potentials are abbreviated, as well as have lower upstroke velocity. TH treated action potentials are prolonged (as compared to control), and have a lower upstroke velocity. Both PD and TH action potentials have an elevated resting membrane potential.

First, we used a simulation model of cardiomyocytes previously described. Simulated cardiac action potential are shown in Figure 5. Compared to comparator simulated from previous publications, simulations from this present study showed PD significantly abbreviated cardiomyocyte action potential durations. We were able to show that TH allowed the prolongation of action potential (AP) duration for both comparator and PD conditions.

#### Simulated Action of TH on ECG

With the simulation of the 3D wedge model, we were able to reproduce a ventricular part of ECG. Supplementary Figure 4 displays a representative frame showing transmural electrical activation in the 3D wedge model. Under NT conditions, the ECG's T wave became inverted as well as abbreviating ST interval. Under TH conditions, ECG simulation showed that T wave inversion became less severe. TH prolonged the ST interval as compared to NT conditions, however moving it toward the control case (Supplementary Figure 4).

## Discussion

This study is the first evaluation of the potential cardiovascular effects of moderate hypothermia in PD patients. We report the effects of dialysate cooling on myocardial blood flow, both at rest and under pharmacological stress, as have demonstrated (through advanced computational modeling of arrythmia formation) that the observed patterns of myocardial perfusion seen with cooling may provide primary protection against cardiac sudden death.

### Feasibility

Whereas, cooling has already been performed in HD patient, it is the first time that cooled dialysate has been used in PD to show impact on microcirculation. Until now, PD cooling was used as a means to profoundly cool experimental animals. This pilot study allows to show that modest cooling of PD dialysate is easy to perform and very well-tolerated. The cooling protocol was simple to deliver and could be incorporated into current delivery systems, cost-free. In contrast with HD, where significant thermal transfer occurs due to the large volume of dialysate which runs in contact with blood over a dialysis treatment, body core temperature seems to be less impacted by the modest reductions in PD fluid temperature we studied. This was also well-tolerated, with no patients reporting they felt cold.

### Myocardial Perfusion and Heterogeneity

Previous studies have demonstrated that HD is characterized by a 30% reduction in regional myocardial blood flow (MBF) (11). Patients in these studies also exhibited PD-induced reductions in MBF despite not having significant occlusive coronary artery disease, suggesting that these changes occur as a result of the decreased coronary flow reserve and microcirculatory effects. While the cardiac effects of HD are well-established, those of PD have not yet been fully studied. We know that PD does influence homeostatic vascular mechanisms (20). Boon et al. (21) showed increased blood pressure during the instillation phases of peritoneal dialysis. More recent studies have shown a hypertensive effect as a direct response to the hyperglycemic, hyperinsulinemic state induced by high glucose concentration containing PD solutions (22). Others investigators have found PD volume related increases in carotid diastolic pressures, and varying carotid baroreceptor sensitivities with differentially buffered PD solutions (23). Over hydration may also alter endothelial function, with reports of an independent correlation between indices of volume status and arterial flow mediated dilatation (24). Our study provides further evidence of the microcirculation dysfunction in PD patients. In our study, myocardial perfusion are quite low and comparable to myocardial perfusion measured during a HD session (11). These results are consistent with previous study where authors showed a same increase of CRF by around 189% in their dialysis patients group (25).

Beyond overall and segmental myocardial perfusion, myocardial heterogeneity has never been studied before in PD patients. Autopsy and experimental studies have demonstrate a reduction in myocardial capillary supply in ESRD patients (26). As previously described, myocardial heterogeneity is a surrogate of endothelial dysfunction and bring additional information on the patients' microcirculation status.

Study of the impact of cooling in PD on MBF and its heterogeneity has not been previously attempted. This study has highlighted that heterogeneity decreased after exposure to cooler dialysate and decreased further under conditions of pharmacological stress. Cooling allowed to significantly more homogenized perfusion. Further, there is increasing basic science evidence that spatial BF heterogeneity in the heart is reduced as the total BF entering the asymmetric coronary arterial structure reduces (27). Therefore, cooling dialysate could be an easy way to protect heart from capillaries heterogeneity and potentially provide cardio-protection.

### Arrhythmia

It is well-known that temperature affects many characteristics of the action potential (AP) wave, especially amplitude, duration, conduction velocity, dispersion of repolarization (28). Our results are consistent with previous reports (29, 30). Indeed, our simulated AP model allows to confirm that TH itself induces an AP duration prolongation. In accordance with actual patient observations (31), our computer model predicted the TH induced QT interval prolongation, as well as T wave amplitude augmentation at V6 lead.

First, our powerful model of simulation underscores the impact of PD on AP and ECG. PD shortens AP and QT, providing more information of the “ischemic” nature of PD. Secondly, TH normalization AP and QT on ECG. Significant pre-clinical experimental evidence corroborates our findings that increase of APD and maintained tissue conductivity are two main antiarrhythmic effects of TH (32). We showed in this study that TH provides potential cardio-protection by strongly dissipating arrhythmic electrical re-entry, even in the presence of simulated structural tissue heterogeneity.

The circadian variation of death in dialysis patients (33), with greater than expected frequencies in the early morning hours, makes a nightly delivered therapy such as PD cooling particularly promising.

### Strength of the Model

One of the strengths of this study is the use of simulation model of AP with cell's model and ECG with a 3D wedge model. Simulations were performed to demonstrate the potential one to one relationship between cooling and ECG features, which were not apparent in patient recordings due to lack of control ECG. Through this simulation, we were able to catch the effects of simulated TH on the ECG which is prolongation of QRS and of the QT interval.

The use of alternative methods such as computer simulations is of great importance. Direct experimental and clinical possibilities for detailed study cardiac arrhythmias in human ventricular myocardium is limited. Multiple ionic currents that govern repolarization are particularly susceptible to hypothermia. An important feature of our model is that all major ionic currents are fitted to data on human ventricular myocytes and expression experiments of human cardiac channels. We can therefore define a simulated model to implement different experimental condition and show potential impact of PD and impact of cooling on cardiac electrophysiology (34).

### Study Limitations

This pilot study of peritoneal dialysate cooling has limitations. The study was not designed to interrogate the driving effect of the cooling intervention given its small sample size. Similarly, although clinical outcomes such as hospitalizations and mortality were tracked in the 9 months period after study completion, it is not adequately powered nor was the intervention delivered for sufficient time to estimate outcomes. We did not measure difference in body core temperature, however we hypothesized infusion of cold peritoneal dialysis leads to cold shock protein synthesis (35). It has been demonstrated that these cold shock protein could protect against ischemia but also regulate electrophysiological properties of the heart, especially cardiac pacemaker activity, and repolarization phase of atrial and ventricular cardiomyocytes (36). Moreover, oral temperature was not precise enough to discriminate difference in body temperature. Although body temperature was monitored all along the cold PD session, we provide only temperature at the start of the normothermic PD session.

## Conclusion

Manipulation of temperature through the use of cooled peritoneal dialysis fluid has significant effects on myocardial perfusion. The observed high resolution changes in perfusion pattern and direct effects on development and propagation of electrical activity through the heart appear to be capable of significantly modifying arrhythmic potential. This proof of principal study provides the means for further optimization of a cooled interventions (in terms of exposure and magnitude) and justifies additional study and development to provide primary protection against cardiac sudden death for patients receiving PD.

## Data Availability Statement

The raw data supporting the conclusions of this article will be made available by the authors, without undue reservation.

## Ethics Statement

The studies involving human participants were reviewed and approved by CRIC: R-16-012; REB approval number: 107280, NCT NCT04394780. The patients/participants provided their written informed consent to participate in this study.

## Author Contributions

SK conceived and performed the study. SK, AS, and CM was involved in protocol development. LH assisted in obtained ethical approval, and assisted SK to perform image analysis. TT assisted in patient recruitment and dialysis delivery. SK and SL performed data analysis and wrote the first manuscript draft. All authors wrote and approved the final manuscript.

## Conflict of Interest

Research funding from Baxter Pr C. McIntyre: Honoraria from Baxter for lecturing. The authors declare that the research was conducted in the absence of any commercial or financial relationships that could be construed as a potential conflict of interest.

## Publisher's Note

All claims expressed in this article are solely those of the authors and do not necessarily represent those of their affiliated organizations, or those of the publisher, the editors and the reviewers. Any product that may be evaluated in this article, or claim that may be made by its manufacturer, is not guaranteed or endorsed by the publisher.
