# Supplementary material for: Therapeutic Hypothermia Reduces Peritoneal Dialysis Induced Myocardial Blood Flow Heterogeneity and Arrhythmia
Source: Front Med (Lausanne). 2021 Jul 30;8:700824. doi: 10.3389/fmed.2021.700824 (PMC8362929; doi:10.3389/fmed.2021.700824)
Supplement: Supplementary file 1 [file Data_Sheet_1.docx]

**S1. Supplementary Methods**

**Computer modelling**

*S1.1 3D human ventricle wedge model:* Assuming diastolic thickness to be 8 mm based on experimental data ([Baillargeon et al., 2014](#_ENREF_1)), the 3D wedge consisted of a 40 mm (X) x 40 mm (Y) x 8 mm (Z, transmural direction from endocardial region to epicardial region) slab as shown in Figure S1, a. The slab was sub-divided into an endocardial layer (12.5%), midmyocardial layer (27.5%), and epicardial layer (60%). Each equally spaced location within the wedge, called a numerical node in the finite difference mesh (see numerical methods below), was assigned the corresponding cardiomyocyte type described in materials and methods section. In addition to electrical inhomogeneity, fibre orientation anisotropy was implemented as described previously ([Göktepe and Kuhl, 2010](#_ENREF_10)). Electrical propagation was simulated using reaction-diffusion equations ([Fenton and Karma, 1998](#_ENREF_7); [Kharche et al., 2014](#_ENREF_13)), where diffusion represented cell-cell electrotonic coupling. Diffusion along the fibres was adjusted to provide a conduction velocity of 0.7 m/s in line with clinical measurements ([Taggart et al., 2000](#_ENREF_18)). Diffusion perpendicular to the fibres was assumed to be 25 times less than along the fibres. Diffusion along transmural direction was assumed to be perpendicular to fibres.

A virtual ECG electrode, to approximately represent the V6 electrode of a 12 lead ECG, was placed 3 cm away from the epicardial surface as shown in Figure S1, b. The virtual ECG lead permitted calculation of a pseudo-ECG signal based on the spatiotemporal electrical patterns in the wedge model ([Gima and Rudy, 2002](#_ENREF_9)).

*S1.2 Implementation of dialysis (ischemia) and TH:* In cardiomyocyte models, the effect of dialysis was implemented as activation of the ATP sensitive cell membrane potassium current, I_KATP_ ([Ferrero et al., 1996](#_ENREF_8)). Extracellular potassium, [K^+^]_o_ was assumed to be elevated to 8 mMol ([Ferrero et al., 1996](#_ENREF_8); [Rodriguez et al., 2006](#_ENREF_17)). Further, the availability of the upstroke sodium and calcium currents, I_Na_ and I_CaL_ respectively, was assumed to be reduced by 25% ([Rodriguez et al., 2006](#_ENREF_17)). At tissue level, ischemia induced reduction of conduction velocity was implemented as a 10 fold reduction of diffusion. In this study, alterations in anisotropy ratio were not considered.

The cell level effects of TH were implemented based on experimental data. The conductances of I_KATP_ and inward rectifier current, I_K1_, were reduced by factors of 1.6 ([McLarnon et al., 1993](#_ENREF_15)) and 1.5 ([Kiyosue et al., 1993](#_ENREF_14)) respectively. TH is also known to reduce time constants of ion channel activation and inactivation. Experimentally, temperature dependence of ion channel gating time constants is quantified using Q_10_ values, which can be directly implemented into the ten Tusscher cardiac cell model. A Q_10_ of 2 was implemented for activation time constant of the slowly activating K^+^ channel, I_Ks_ ([Faber et al., 2007](#_ENREF_6)). For the HERG channel (I_Kr_) activation and inactivation, Q_10_ values of 2.1 and 2.6 respectively ([Vandenberg et al., 2006](#_ENREF_21)) were implemented. The I_Na_ current’s activation and inactivation time constants’ Q_10_ values are known to be 2 and 2.2 respectively ([Collins and Rojas, 1982](#_ENREF_4); [O'Hara et al., 2011](#_ENREF_16)). The Q_10_ for I_CaL_ current’s inactivation time constant was implemented as 2.1 ([Kiyosue et al., 1993](#_ENREF_14)). At tissue level, the effect of TH was implemented as the same diffusive coupling as in the control (non-dialysed and untreated using TH) case.

*S1.3 Pacing protocols for ECG and scroll waves:* In simulations designed to produce pseudo ECG, the wedge was simulated at the endocardial surface at a pacing cycle length of 850 ms ([Fabbri et al., 2017](#_ENREF_5)), and the waves permitted to propagate transmurally. Simulations were also performed to assess whether dialysis as an ischemia could be an electrophysiological substrate for persistent tachycardia, and whether TH could abrogate tachycardia life spans. To do so, in wedges that represented either dialysed or TH treated, a scroll wave using a phase distribution method previously developed ([Biktashev and Holden, 1998](#_ENREF_3); [Kharche et al., 2015](#_ENREF_12)) was induced at the centre of the wedge and permitted to evolve for a span of 3 seconds. If the spiral wave self-terminated or meandered out of the wedge, then it was defined as self-termination, otherwise it was defined as persistent.

*S1.4 Numerical methods:* The 3D wedge was discretized as a uniform finite difference grid with a spatial resolution of 0.1 mm. A time step of 0.1 ms was used in all simulations, cell as well as 3D. In the 3D spatial simulations, a backward difference formula based implicit method was used to solve the reaction (ordinary differential equations describing the cell’s electrophysiological dynamics) part, while a second order finite difference explicit method was used to solve the diffusion (partial differential equation describing electrical wave propagation) part. These methods are based on robust libraries ([Balay, 2012](#_ENREF_2); [Hindmarsh et al., 2005](#_ENREF_11)) and implemented into our in house mechanistic simulator called PM^3^ (Precision Medicine using Mathematical Modelling). PM^3^ is a high performance computing application.

**Table 1S :Patients’ characteristics: anthropometrics values, medical history and medications.**

Values are expressed as mean ± Sd or number (%).

| **Characteristic** | |
| --- | --- |
| Age (years) | 60 ± 7 |
| Female (%) | 4 (57) |
| Weight (Kg) | 77 ± 23 |
| Height (cm) | 163 ± 11 |
| BMI (kg/m^2^) | 29.2 ± 6.4 |
| Urinary residual Volume (mL) | 400 ± 300 |
| **Medical history** | |
| PD vintage (months) | 23.6 ± 17.6 |
| Ischemic heart disease (%) | 1 (14) |
| Current or ex-smoker (%) | 2 (29) |
| Peripheral vascular disease (%) | 1 (14) |
| **Medication** | |
| Treated hypertension (%) | 6 (86) |
| RAAS antagonist (%) | 5 (71) |
| β-blocker (%) | 4 (43) |
| Statin use (%) | 5 (71) |
| Phosphate binder *Calcium containing* (%) | 5 (71) |
| Phosphate binder *Non calcium* (%) | 1 (14) |
| Erythropoiesis-stimulating agent (%) | 6 (86) |
| Vitamin D analog (%) | 4 (43) |

**Legends to figures**

**Figure S1. 3D wedge model anatomy**. The model is a 3D slab representing a large part of the left human ventricle. It is 40 mm x 40 mm, and has a transmural thickness of 8 mm. The slab is transmurally sub-divided into an epicardial slice, a midmyocardial (or sub-endocardial) slice, and a thicker epicardial slice. In addition, fibre directions are assigned to each location in accordance with previous imaging based findings (Kuhl). A virtual ECG electrode was placed 3 cm outside of the wedge as shown. The electrode permitted calculation of the pseudo-ECG based on electrical activity in the wedge.

**Figure S2. Composition of the structural ischemic heterogeneity**. Epicardial and transmural views are shown. Top row shows the heterogeneity’s surface, while bottom row shows the heterogeneity when sliced in the plane of the page. The structural ischemic heterogeneity consists of a border zone (BZ) where significant electrophysiological parameter gradients are present. The border zone (BZ) encloses a central ischemic zone (CIZ) that has ischemic electrophysiological properties but is devoid of parameter gradients.

**Figure 3S: Comparison of core temperature (A) mean arterial blood pressure (MAP) (B) and Hear rate (C) between normothermic (NT) and therapeutic hypothermia (TH) conditions.**

A. There is no difference in core temperature between NT and TH. B. MAP was significantly increase in NT condition, p<0.05 C. There is no significant difference in heart rate between NT an dTH condition. We performed non parametric paired test between NT and TH condition.

**Figure 4S: Impact of peritoneal dialysis (PD) and therapeutic hypothermia (TH) on pECG L**

Effects of PD and TH on simulated ECG (pECG). A. Representative frames showing transmural electrical activation in the 3D wedge model. B. ECG traces recorded for simulations based on control (NT), TH, PD (ischemia), and PD electrophysiology are shown. Color coding is given in the legend.

**Figure 5S: Assessment of tachycardia persistence in the presence of ischemic structural heterogeneity.**

A. Epicardial view of the 3D wedge model showing location of ischemic structural heterogeneity. Also shown are the initial conditions that induce re-entry using our previously developed phase distribution method. B. pECG for the duration of the simulated electrical activity in the 3D wedge under control (gray), TH (black), PD (red), and PD with TH (blue) cases. Re-entrant activity self terminated in all cases except in the PD (i.e. ischemic) case, where it persisted for the duration of the simulated time of 6 s, and therefore was considered persistent.

**Figure 1S**


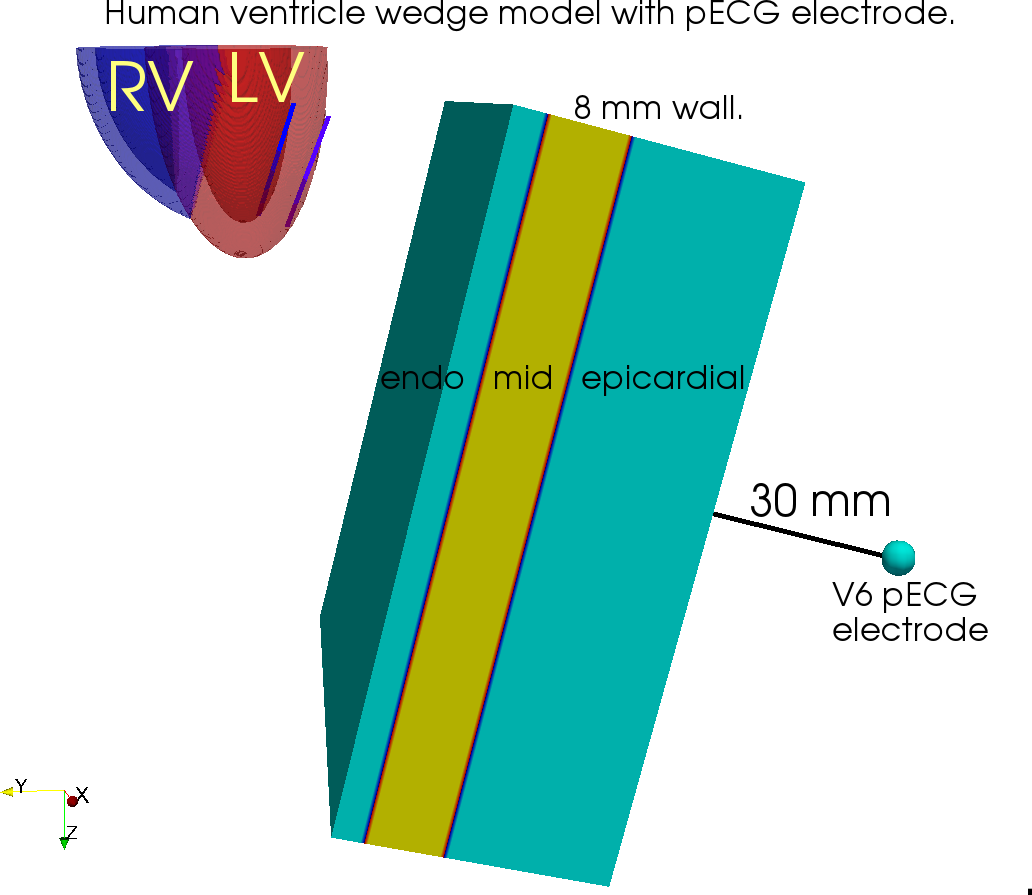


**Figure 2S**


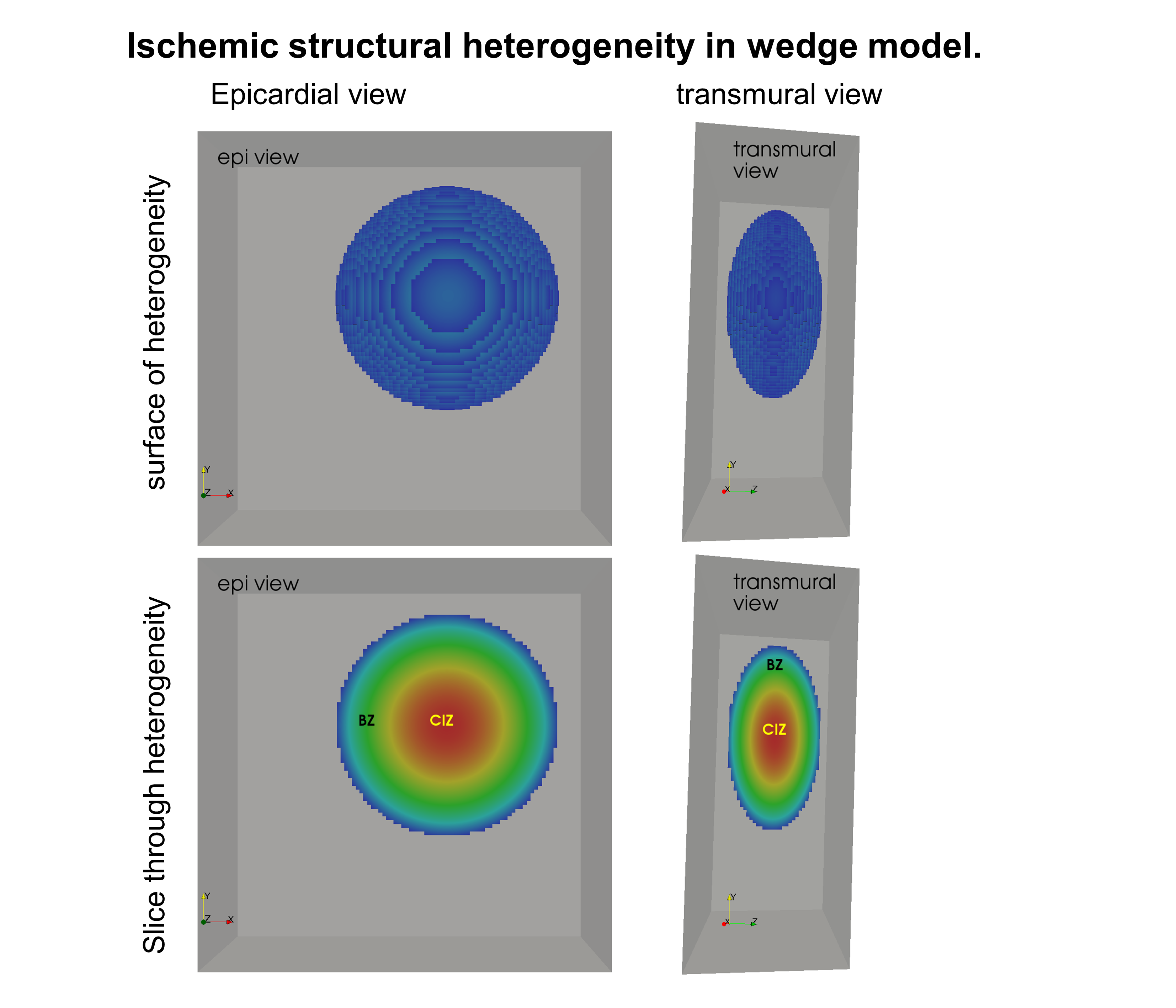


**Figure 3S**

**Figure 4S**


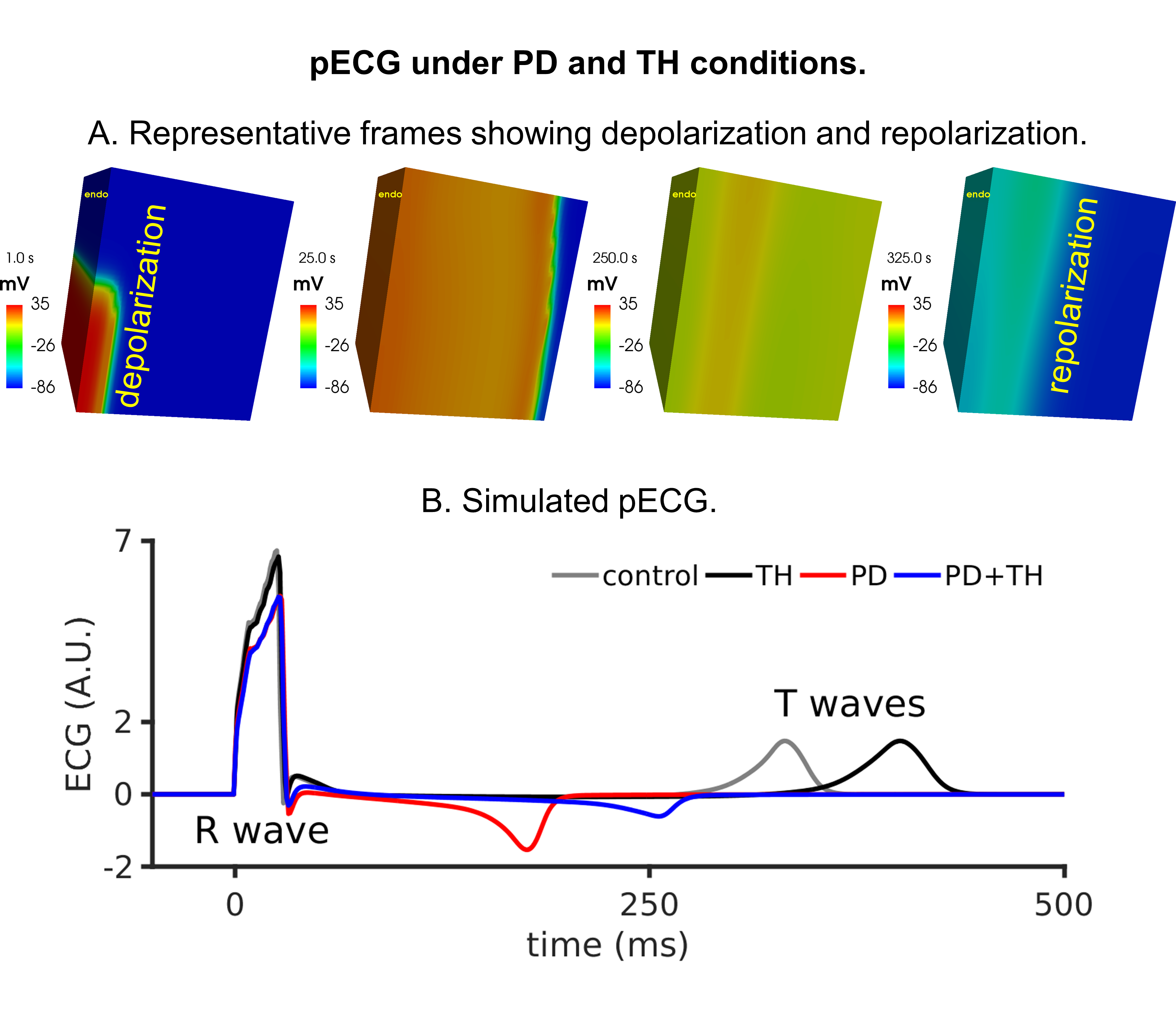


**References**

**Baillargeon, B., Rebelo, N., Fox, D.D., Taylor, R.L., and Kuhl, E. (2014). The Living Heart Project: A robust and integrative simulator for human heart function. European journal of mechanics A, Solids *48*, 38-47.**

**Balay, S., Brown, J, Buschelman, K, Eijkhout, Knopp, WD (2012). PETSc Users Manual (Argonne National Laboratory: Argonne National Laboratory).**

**Biktashev, V.N., and Holden, A.V. (1998). Reentrant waves and their elimination in a model of mammalian ventricular tissue. Chaos *8*, 48-56.**

**Collins, C.A., and Rojas, E. (1982). Temperature dependence of the sodium channel gating kinetics in the node of Ranvier. Q J Exp Physiol *67*, 41-55.**

**Fabbri, A., Fantini, M., Wilders, R., and Severi, S. (2017). Computational analysis of the human sinus node action potential: model development and effects of mutations. J Physiol *595*, 2365-2396.**

**Faber, G.M., Silva, J., Livshitz, L., and Rudy, Y. (2007). Kinetic properties of the cardiac L-type Ca2+ channel and its role in myocyte electrophysiology: a theoretical investigation. Biophys J *92*, 1522-1543.**

**Fenton, F., and Karma, A. (1998). Vortex dynamics in three-dimensional continuous myocardium with fiber rotation: Filament instability and fibrillation. Chaos *8*, 20-47.**

**Ferrero, J.M., Saiz, J., Ferrero, J.M., and Thakor, N.V. (1996). Simulation of action potentials from metabolically impaired cardiac myocytes. Role of ATP-sensitive K+ current. Circ Res *79*, 208-221.**

**Gima, K., and Rudy, Y. (2002). Ionic current basis of electrocardiographic waveforms: a model study. Circ Res *90*, 889-896.**

**Göktepe, S., and Kuhl, E. (2010). Electromechanics of the heart: a unified approach to the strongly coupled excitation–contraction problem. Computational Mechanics *45*, 227-243.**

**Hindmarsh, A.C., Brown, P.N., Grant, K.E., Lee, S.L., Serban, R., Shumaker, D.E., and Woodward, C.S. (2005). SUNDIALS: Suite of Nonlinear and Differential/Algebraic Equation Solvers. ACM Transactions on Mathematical Software *31*, 33.**

**Kharche, S.R., Biktasheva, I.V., Seemann, G., Zhang, H.G., and Biktashev, V.N. (2015). A Computer Simulation Study of Anatomy Induced Drift of Spiral Waves in the Human Atrium. Biomed Res Int 2015; 2015: 731386 Epub 2015/10/26.**

**Kharche, S.R., Stary, T., Colman, M.A., Biktasheva, I.V., Workman, A.J., Rankin, A.C., Holden, A.V., and Zhang, H. (2014). Effects of human atrial ionic remodelling by beta-blocker therapy on mechanisms of atrial fibrillation: a computer simulation. Europace *16*, 1524-1533.**

**Kiyosue, T., Arita, M., Muramatsu, H., Spindler, A.J., and Noble, D. (1993). Ionic mechanisms of action potential prolongation at low temperature in guinea-pig ventricular myocytes. J Physiol *468*, 85-106.**

**McLarnon, J.G., Hamman, B.N., and Tibbits, G.F. (1993). Temperature dependence of unitary properties of an ATP-dependent potassium channel in cardiac myocytes. Biophys J *65*, 2013-2020.**

**O'Hara, T., Virag, L., Varro, A., and Rudy, Y. (2011). Simulation of the undiseased human cardiac ventricular action potential: model formulation and experimental validation. PLoS Comput Biol *7*, e1002061.**

**Rodriguez, B., Trayanova, N., and Noble, D. (2006). Modeling cardiac ischemia. Ann N Y Acad Sci *1080*, 395-414.**

**Taggart, P., Sutton, P.M., Opthof, T., Coronel, R., Trimlett, R., Pugsley, W., and Kallis, P. (2000). Inhomogeneous transmural conduction during early ischaemia in patients with coronary artery disease. J Mol Cell Cardiol *32*, 621-630.**

**Ten Tusscher, K.H., Bernus, O., Hren, R., and Panfilov, A.V. (2006). Comparison of electrophysiological models for human ventricular cells and tissues. Prog Biophys Mol Biol *90*, 326-345.**

**ten Tusscher, K.H., Noble, D., Noble, P.J., and Panfilov, A.V. (2004). A model for human ventricular tissue. Am J Physiol Heart Circ Physiol *286*, H1573-1589.**

**Vandenberg, J.I., Varghese, A., Lu, Y., Bursill, J.A., Mahaut-Smith, M.P., and Huang, C.L. (2006). Temperature dependence of human ether-a-go-go-related gene K+ currents. Am J Physiol Cell Physiol *291*, C165-175.**
